# Peer Review of “The Impact of Rural Alimentation on the Motivation and Retention of Indigenous Community Health Workers in India: Qualitative Study”

**DOI:** 10.2196/70808

**Published:** 2025-01-23

**Authors:** Sanjeev Kumar Thalari

**Affiliations:** 1 Department of Management Studies & Research Center, CMR Institute of Technology, Bangalore, India

**Keywords:** rural alimentation, community health workers, motivation, retention, rural health, rural nutrition, workforce

*This is the peer-review report for “The Impact of Rural Alimentation on the Motivation and Retention of Indigenous Community Health Workers in India: Qualitative Study.”*

## Round 1 Review

### General Comments

This paper [1] has given the impression that the researcher has done thorough homework before starting the research and it is evident in the paper. Case methodology and thematic analysis are a few of the approaches that depict the quality of the paper. Overall, as a reviewer, it is my opinion that the research paper is of quality.

### Specific Comments

1. A few more factors like government initiatives should be included in studying the impact on the motivation and retention of community health workers.

### Major Comments

1. I feel that the analysis also can include education as a parameter.

2. The thematic analysis is one of the strengths of this research and is appreciated.

### Minor Comments

1. Common wording should be used in every section of the paper, like qualitative case research methodology and qualitative case research.
